# Thoracoscopic Sympathicotomy vs Sympathectomy in Primary Hyperhidrosis

**DOI:** 10.5812/traumamon.6335

**Published:** 2012-07-31

**Authors:** Hassan Ali Mohebbi, Shaban Mehrvarz, Shahram Manoochehry

**Affiliations:** 1 Trauma Research Center, Baqiyatallah University of Medical Sciences, Tehran, IR Iran; 2 Department of General Surgery, Faculty of Medicine, Baqiyatallah University of Medical Sciences, Tehran, IR Iran

**Keywords:** Sympathectomy, Sympathicotomy, Hyperhidrosis, Patient Satisfaction

## Abstract

**Background:**

Primary hyperhidrosis (P.H.H.) is characterized by excessive sweating in certain parts of body. It’s estimated prevalence is 0%-6.1% in different populations. In Asian population its prevalence is around 3%. In 57% of cases, there is a positive family history.

**Objectives:**

To evaluate and compare the early and late satisfaction, outcomes and complications of thoracoscopic sympathectomy and sympathicotomy in the treatment of primary hyperhidrosis.

**Materials and Methods:**

From April 2007 to January 2011, we prospectively treated 60 primary hyperhidrosis patients via thoracoscopic surgery. The first 30 patients underwent sympathectomy and the next 30 patients underwent sympathicotomy. We evaluated early and late satisfactions, outcomes and complications on the first visit (5-8days) following surgery and 12 months after surgery, for all patients.

**Results:**

The mean operative time was 66.3 minutes in sympathicotomy group and 110.8 minutes in sympathectomy group (P < 0.001). There were no significant differences between the two groups in overall early and late satisfaction, gustatory sweating, pompholyx and post-operative pain. There was comparatively less early and late compensatory sweating (C.S.), and other adverse influences of C.S. in the sympathicotomy group.

**Conclusions:**

Because of shorter operative time, less C.S. and less adverse influence of C.S., sympathicotomy seems a better treatment for primary hyperhidrosis, compared with sympathectomy.

## 1. Background

Primary hyperhidrosis (P.H.H.) is characterized by excessive sweating in specific areas of the body. It’s estimated prevalence is 0%-6.1% in different populations. In Asian population its prevalence is around 3%. In 57% of cases, there is a positive family history (1). Hyperhidrosis is most prominent in palms, axillae, feet and face. It affects patient’s work, education and other social relationships. It causes psychological problems for patients (2). Medical treatments are not effective and response is usually transient. Surgical therapy is effective and gives better satisfaction. It is based on interruption of sympathetic impulses transmission from ganglia to eccrine sweat glands. Thoracoscopic sympathectomy was first described in 1942 by Hughs; and since the 1980s, it has been recognized as the preferred method of the treatment for primary hyperhidrosis (3).

Compensatory sweating (C.S) is the most common side effect of this surgical procedure, followed by gustatory sweating (G.S.). Other complications include Horner’s syndrome (myosis, ptosis, anhidrosis) and pompholyx or dyshidrotic eczema (a palmar eczema characterized by maculopapules and vesicles because of severe palmar anhidrosis and dry skin). Some studies have reported the possibility of decreasing complications and side effects in sympathicotomy compared to sympathectomy, (4-6) while some studies do not accept this (7, 8).

## 2. Objectives

The aim of this study was to compare the outcomes, satisfactions and complications after thoracoscopic sympathectomy with thoracoscopic sympathicotomy for the treatment of primary hyperhidrosis.

## 3. Patients and Methods

From April 2007 to January 2011 we included 60 patients diagnosed for P.H.H. of hands, axillae and feet (or a combination of these) who had moderate to severe disease in at least one of these areas (score 3 or more, on a 1-5 scale). All patients had tried medical treatments at least once before, and were not satisfied or they had aborted the treatment because of the adverse effects. All surgeries were done at the Baqiyatallah Hospital (Tehran, IR Iran) by one thoracic surgeon (H.A.M.). All patients were perfectly educated regarding the side effects of treatment before undergoing surgery. All the patients signed an informed consent sheet about the study. The medical ethics committee of the Baqiyatallah University of Medical Sciences approved this prospective clinical trial (which was proposed as a thesis). The first 30 patients were operated by thoracoscopic bilateral sympathectomy and the next 30 patients were operated by thoracoscopic bilateral sympathicotomy.

### 3.1. Data Collection

We used a questionnaire which included patients’ demographics, severity of disease, surgical details, and complications, outcomes and satisfaction. Short-term (early) evaluations were done at the first visit following surgery (5-8 days after surgery). Long-term (late) evaluations were done at a visit or via telephone inquiry one year after surgery.

### 3.2. Statistical Analysis

Quantitative variables were described using index of centralization and dispersion: mean, standard and range deviation. Normality hypothesis of said variables was contrasted using the Kolmogorov-Smirnov test for only one sample. Qualitative variables analyzed the absolute frequency of the appearance of each of the categories such as relative frequencies. The comparison of categorical data was done using the chi-square test or Fisher’s exact test when necessary. Quantitative variables and ordinal qualitative variables were compared among the groups using Mann-Whitney test. The statistical signiﬁcance level was set at P < 0.05. We made use of the statistical software package SPSS version 18 for windows for the analysis.

### 3.3. Operative Technique

**Figure 1:**
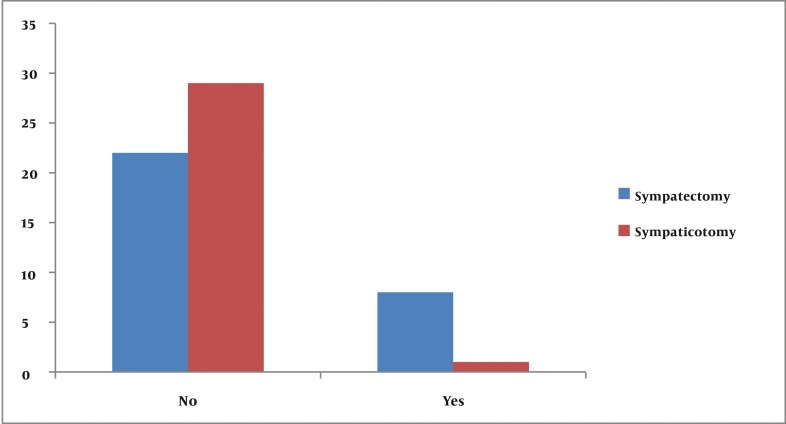
Early adverse influence on C.S. on life

All surgeries were performed under general anesthesia with electrocardiography and pulse oxymetery monitoring. In almost all the patients, surgery was done in semi –fowler position with both hands abducted 90^º^. In these patients thoracoscopy was done with CO_2_ insufflations. The exception was in a few first patients with sequential lateral position and without CO_2_ insufflations. Thoracoscopic sympathectomy was done through a 5mm throcar inserted in the third intercostal space (I.C.S.) at mid axillary line, and a 10 mm throcar in the forth I.C.S. at anterior axillary line. Thoracoscopic sympathicotomy was performed through a 10 mm port in the third I.C.S. at mid axillary line. Sympathectomies and sympathicotomies were done at T2-T3 for palmar hyperhidrosis (H.H), at T3-T4 for axillary H.H. plantar H.H. alone or in combination, and at T2-T3-T4 for palmar-axillary H.H. with or without plantar H.H (8). Figure 1 shows both surgical procedures.

### 3.4. Variables

Age, sex, type of H.H. and family history were analyzed. The following variables were defined: Patient’s satisfaction was defined as percent of patient’s satisfaction from the results of surgery and improving of H.H. Severity of H.H. was defined on a 1-5 scale as: 1 normal, 2 sometimes having moisture, 3 often having moisture, 4 sometimes dripping and 5 often dripping (8). Compensatory sweating (excessive sweating in other parts of body) was classified as: mild: not bothering, moderate: sometimes bothering, severe: often bothering (8). Gustatory sweating (facial sweating during eating or chewing) was classified as: mild: not bothering, moderate: sometimes bothering, severe: often bothering (8). Post-operation pain was classified according to the Visual Analog Pain Scale (VAPS) from 0 to 10.

## 4. Results

Patients' demographic data is summarized in Table 1. There were no significant statistical differences in the severity of palmar, plantar and axillary hyperhidrosis between the groups (Table 2). There were two lung injuries while inserting ports due to some adhesions of pleura in the sympathectomy group and one in the sympathicotomy group. Chest tubes were inserted for these patients. There was no conversion to open surgery. Mean operative time in the sympathectomy group was significantly longer than that of the sympathicotomy group, (110.87 ± 13.31 min vs 62.33 ± 17.96 min respectively) (P < 0.001).

**Table 1:** Patients Demographics

Variables	All	Sympathectomy group	Sympathicotomy group	*P* Value
Age (mean ± SD)	25.93 ± 6.49	25.47 ± 6.95	26.4 ± 6.08	0.582
Sex				0.584
Male	46 (76.7 %)	20 (66.7 %)	26 (86.7 %)	
Female	14 (23.3 %)	10 (23.3 %)	4 (13.3 %)	
Family history	29 (48.88 %)	14 (46.66 %)	15 (50 %)	0.98

**Table 2:** Severity of H.H. in Both Groups

Severity of H. (1-5)	Sympathectomy group	Sympathicotomy Group	*P* Value
Palmar H.H.			0.940
Nor (1)	0 (0 %)	0 (0 %)	
Mild (2)	0 (0 %)	1 (3.3 %)	
Mod (3)	0 (0 %)	2 (6.7 %)	
Severe(4)	9 (30 %)	14 (46.7 %)	
Very Severe(5)	21 (70 %)	13 (43.3 %)	
Axillary H.H.			0.504
Nor (1)	7 (23.3 %)	6 (20 %)	
Mild(2)	4 (13.3 %)	4 (13.3 %)	
Mod(3)	4 (13.3 %)	5 (16.7 %)	
Severe(4)	6 (20 %)	6 (20 %)	
Very Severe(5)	9 (30 %)	9 (30 %)	
Plantar H.H.			0.878
Nor (1)	1 (33 %)	0 (0 %)	
Mild(2)	0 (0 %)	1 (3.3 %)	
Mod(3)	6 (20 %)	3 (10 %)	
Severe(4)	8 (26.7 %)	13 (43.3 %)	
Very Severe(5)	15 (50 %)	13 (43.3 %)	

**Figure 2:**
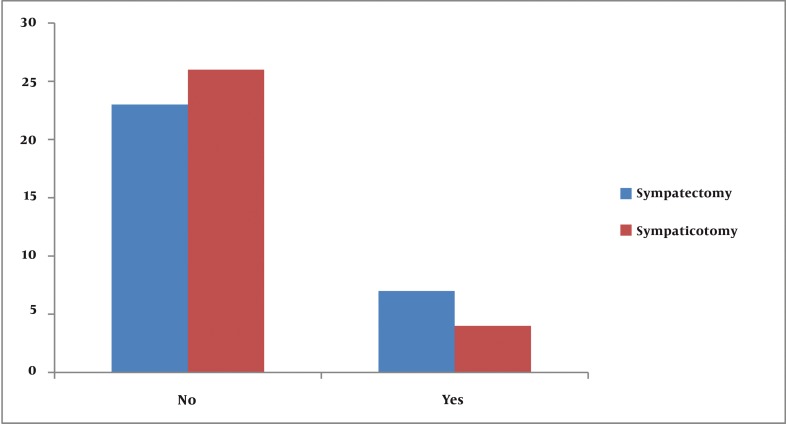
Late adverse influence of C.S. on life

**Figure 3:**
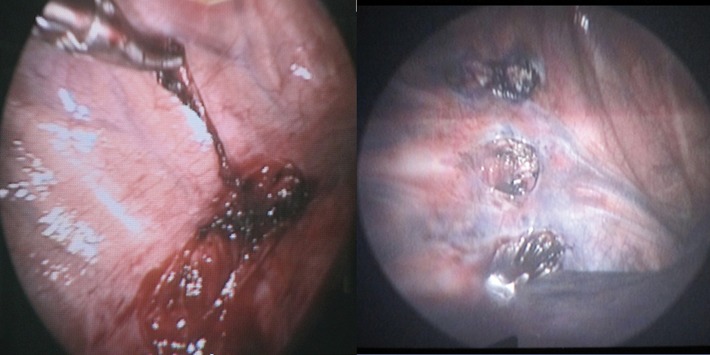
Photos of Surgical Procedures. Left: Sympathectomy. Right: sympathicotomy

The overall incidence of both early and late compensatory sweating was 90% in the sympathectomy group and 73.3% in the sympathicotomy group. (P = 0.001) Early and late satisfactions from surgery (according to sites of body) are summarized in Table 3. Six patients (20%) in the sympathectomy group and 4 patients (13.3%) in the sympathicotomy group had gustatory sweating (G.S.). There was no significant difference in G.S. between the two groups. (P = 0.652) There was no significant difference between the two groups in the incidence of pompholyx (Dyshidrotic eczema). Table 4 shows this data. Mean post-operative pain numbers in sympathectomy and sympathicotomy groups were 6.25 ± 2.31and 5.33 ± 1.84 (V.A.P.S.) respectively in early evaluations (P = 0.101). In late evaluations mean pain numbers were 2.3 ± 1.03 and 1.93±0.98 (P = 0.702). There were no significant differences between early and late adverse effects of C.S. on life in groups. This data is shown in figures 1 and 2. There were no other post-operative complications such as pneumothorax, hemothorax, Horner’s syndrome and recurrence in our patients during follow-up period (Figure 3).

**Table 3:** Satisfaction (early and late) in Groups

Sites	Satisfaction in sympathectomy group mean ± SD	Satisfaction in sympathicotomy group mean ± SD	*P* Value
**Early**			
Hands	98% ± 9	99% ± 3	0.461
Axillae	84% ± 2	93% ± 8	0.090
Feet	43% ± 32	60% ± 29	0.033
**Late**			
Hands	97% ± 3	94% ± 4	0.028
Axillae	83% ± 26	86% ± 9	0.947
Feet	40% ± 33	39% ± 20	0.868

**Table 4:** Incdence of Pompholyx after Surgery

Evaluations	Sympathectomy group	Sympathicotomy group	*P* Value
Early	3 (10.00 %)	1 (3.33 %)	0.101
Late	6 (20.00 %)	4 (13.33 %)	0.702

## 5. Discussion

Previous studies have reported that thoracoscopic sympathectomy cures or reduces essential hyperhydrosis with a success rate and satisfaction over 90 % (2, 6, 8, 9). Some articles have reported that the degree of satisfaction after surgery decreases with time and type of hyperhidrosis (palmar, axillary, plantar), while there is controversy about the role of limiting sympathectomy on the degree of satisfaction (6, 10-12). In our study, the overall early and late satisfaction rate between the two groups were not significantly different. The satisfaction rate in all the sites (hands, axillae and feet respectively) decreased with time. Additionally, the satisfaction rate decreased with sites of hyperhidrosis (palmar, axillary, plantar respectively) in a decreasing pattern, and these results are similar to previous reports.

There are many articles, reporting no recurrences during their follow-up period (13, 14), while others reported 3%─8.2% recurrence rates (2, 4, 15). We saw no recurrences in either of the groups during the 12-month follow-up period. This may be partly related to surgical precision in finding and severing interganglionic tracts and Kuntz's fibers (16) leading to longer operation time compared to the other reports (4, 8). Lack of recurrence in our patients may also be partly because of shorter follow-up period of this trial, although one of the reports with 7.45% recurrences had a follow up period of similar duration (i.e. 12 months) like our study (15). Compensatory sweating in sites such as the face, chest, back, abdomen and legs are reported from 9% to 100% in different articles (7, 8). C.S. is sometimes severe enough to nullify the outcome of surgery (6, 7).

Several authors described fewer C.S. and G.S by limiting the manipulation, rather than the type of sympathetic disconnection (sympathectomy or sympathicotomy) (16, 17), while others reported fewer side effects with sympathicotomy (5, 18). Rodrigues reported no relation between the levels of sympathicotomy and C.S. (6). Gossot reported that both sympathicotomy (instead of sympathectomy) and limiting the level of procedure would lower C.S. (19). In this trial there were less early and late C.S. in the sympathicotomy group than the sympathectomy group. This is in agreement with some of the above mentioned reports. Horner's syndrome (H.S.) may occur at a mean rate of 0.3% to 0.7% in patients undergoing thoracic sympathetic manipulations (8). We had no H.S. in our patients. This may be because of levels of sympathetic manipulations in our patients which were from T2 downward. Adverse influence of C.S. on life was less in both early and late evaluations in the sympathicotomy group. These are partly because of the fact that there was no case of severe C.S. in the sympathicotomy group but we had some cases of severe C.S. in the sympathectomy group.

The rate of G.S. is from 5% to 50% in different reports (1, 2, 5, 6, 15). The rates of G.S.in this study are moderate and similar to previous reports. There were not statistically significant differences between two groups in this regard. There was no significant difference between groups in post-operative pain, although we had inserted two trocars in sympathectomy group but the additional trocar comparing with the sympathicotomy group was a 5 mm trocar and this may describe the rather equal post-operative pain in the two groups. There were both over all less C.S. and less adverse influence on life in the sympathicotomy group than in sympathectomy group. There were no significant differences in overall satisfaction, G.S. pompholyx and post-operative pain between the groups. There was a significant shorter mean operation time in the sympathicotomy group. For reasons mentioned above, sympathicotomy is a better procedure for the treatment of hyperhidrosis, compared with sympathectomy.
